# Evidence of a Large-Scale Functional Organization of Mammalian Chromosomes: Authors' Reply

**DOI:** 10.1371/journal.pbio.0050128

**Published:** 2007-05-15

**Authors:** Sagiv Shifman, Jordana Tzenova Bell, Richard R Copley, Martin S Taylor, Richard Mott, Jonathan Flint, Robert W Williams

We agree with Petkov and colleagues that including the BXD lines (“new BXD”), which were derived from advanced intercross lines (AIL), increases the map length in the recombinant inbred (RI) lines. As we note in our paper “One BXD subset was developed using advanced intercross populations and therefore contains approximately 1.7 times more recombinants per line”[1]. If the “new BXD” set were analyzed by itself, an AIL-specific map expansion factor should be applied [2]. However since our motivation was to obtain an accurate estimation of the relative rates of recombination in different regions of the genome, we analyzed all of the RIs together to increase the number of recombination events and the overall density of polymorphic markers. This increased our sensitivity, for example, to identify regions of extremely low and high recombination rates.

There are a number of explanations as to why the map length should differ from that expected on the basis of the Haldane-Waddington equation [3,4].

The estimated map distance of the RIs depends also on the marker density [2] and on the number of inbred generations [5], factors that act to reduce the map length relative to the Haldane-Waddington theoretical equation (which assumes a fully inbred line). It has been hypothesized that “the apparent genetic interference of the accumulated generations (e.g., RIs) differs from the genetic interference acting in each meiosis and that commonly used map functions lead to reduced map distance estimates in the advanced designs” [5]. Consequently, it is not clear that the observed can be explained solely in the way suggested by Petkov et al.

## References

[pbio-0050128-b001] Shifman S, Bell JT, Copley RR, Taylor MS, Williams RW (2006). A high-resolution single nucleotide polymorphism genetic map of the mouse genome. PLoS Biol.

[pbio-0050128-b002] Winkler CR, Jensen NM, Cooper M, Podlich DW, Smith OS (2003). On the determination of recombination rates in intermated recombinant inbred populations. Genetics.

[pbio-0050128-b003] Haldane JB, Waddington CH (1931). Inbreeding and linkage. Genetics.

[pbio-0050128-b004] Martin OC, Hospital F (2006). Two- and three-locus tests for linkage analysis using recombinant inbred lines. Genetics.

[pbio-0050128-b005] Teuscher F, Guiard V, Rudolph PE, Brockmann GA (2005). The map expansion obtained with recombinant inbred strains and intermated recombinant inbred populations for finite generation designs. Genetics.

